# Inner structure- and surface-controlled hollow MnO nanocubes for high sensitive MR imaging contrast effect

**DOI:** 10.1186/s40580-020-00227-6

**Published:** 2020-05-12

**Authors:** Aastha Kukreja, Byunghoon Kang, Seungmin Han, Moo-Kwang Shin, Hye Young Son, Yuna Choi, Eun-Kyung Lim, Yong-Min Huh, Seungjoo Haam

**Affiliations:** 1 Department of Chemical and Biomolecular Engineering, Yonsei University, 50 Yonsei-ro, Seodaemun-gu, Seoul, 03722 Republic of Korea; 2 BioNanotechnology Research Center, Korea Research Institute of Bioscience and Biotechnology (KRIBB), 125 Gwahak-ro, Yuseong-gu, Daejeon, 34141 Republic of Korea; 3 Division of Cardiothoracic Surgery, Department of Surgery, University of Arizona, Tucson, AZ 85724 USA; 4 Department of Radiology, College of Medicine, Yonsei University, 50-1 Yonsei-ro, Seodaemun-gu, Seoul, 03722 Republic of Korea; 5 Severance Biomedical Science Institute, College of Medicine, Yonsei University, 50-1 Yonsei-ro, Seodaemun-gu, Seoul, 03722 Republic of Korea; 6 YUHS-KRIBB Medical Convergence Research Institute, 50-1 Yonsei-ro, Seodaemun-gu, Seoul, 03722 Republic of Korea; 7 Department of Nanobiotechnology, KRIBB School of Biotechnology, University of Science and Technology (UST), 217 Gajeong-ro, Yuseong-gu, Daejeon, 34113 Republic of Korea

**Keywords:** Hollow nanostructure, Ligand encapsulation and exchange, Manganese oxide nanocube, MR imaging, T1 contrast agent

## Abstract

Manganese oxide (MnO) nanocubes were fabricated and their surface were modified by ligand encapsulation or ligand exchange, to render them water-soluble. And then, MnO formed the hollow structure by etching using acidic solution (phthalate buffer, pH 4.0). Depending on the ligand of the MnO surface, it increases the interaction between MnO and water molecules. Also, the hollow structure of MnO, as well as the ligand, can greatly enhance the accessibility of water molecules to metal ions by surface area-to-volume ratio. These factors provide high R1 relaxation, leading to strong T1 MRI signal. We have confirmed T1-weighted MR contrast effect using 4-kinds of MnO nanocubes (MnOEn, MnOEnHo, MnOEx and MnOExHo). They showed enough a MR contrast effect and biocompatibility. Especially, among them, MnOExHo exhibited high T1 relaxivity (r1) (6.02 mM^−1^ s^−1^), even about 1.5 times higher sensitivity than commercial T1 MR contrast agents. In vitro/in vivo studies have shown that MnOExHo provides highly sensitive T1-weighted MR imaging, thereby improving diagnostic visibility at the disease site.
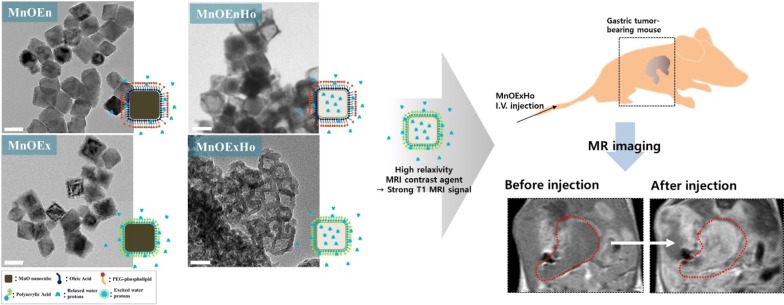

## Introduction

Magnetic resonance imaging (MRI) is not only able to provide anatomical images with significant spatial resolution and depth, but also attracts more and more attention due to its non-radioactive and non-invasive properties [1–5]. Two types of contrast agents are used for MRI scans. Negative contrast or T2-weighted magnetic resonance (MR) images are generated by superparamagnetic iron oxide nanoparticles, reducing the spin–spin relaxation time of the water [6–9]. Positive contrast or proton MR images are generated by T1-based contrast agents such as manganese oxide (MnO) nanoparticles or gadolinium (Gd^3+^) to reduce spin–lattice relaxation time of nearby water molecules, resulting in the brightening of the voxel [10–13]. Tremendous research has led to significant advancements in technology and the controlled construction of nanomaterials. Chemical, physical, or biological methods can be used for the synthesis of nanomaterials, allowing for accurate control of their shape, composition, size and surface characteristics [14–18]. These parameters can be easily tailored to tune the physical and chemical properties of nanomaterials. Through decades of research, a diverse approach has been developed for the synthesis of MnO nanoparticles [10, 19–27]. Despite the increased interest, T1-based MRI is relatively limited due to ratiometric complications and low signal intensities. In addition, to influence the relaxation time, T1 contrast agent needs to interact directly with the surrounding water protons [28–30]. In case of nanospheres, only ions that are exposed on the surface are effective. Reducing the particle size on the basis of the same concentration of ions that make up the particle can provide a larger surface area. Additionally, by transforming the particles to the hollow structures, the particle surface percentage can be increased, which can, in turn, provide higher relaxivity. Hollow particles can offer high specific area and have excellent penetration and permeability. The volumetric capacity is quite high, which allows access to more water molecules to improve MR contrast [31, 32]. In addition, ligand exchange can make the surface hydrophilic, which further enhances the contrast compared to bilayer coated particles. Hydrophobic inner coating can hinder moisture penetration and reduce spin effect [33–36]. Until now, several hollow MnO-based nanoparticles have been developed, including MnO particles loaded with iron oxide nanoparticles or drug molecules [37–39]. Despite this development, hollow MnO particles have not been optimized for low relaxation and maximum contrast enhancement.

**Fig. 1:**
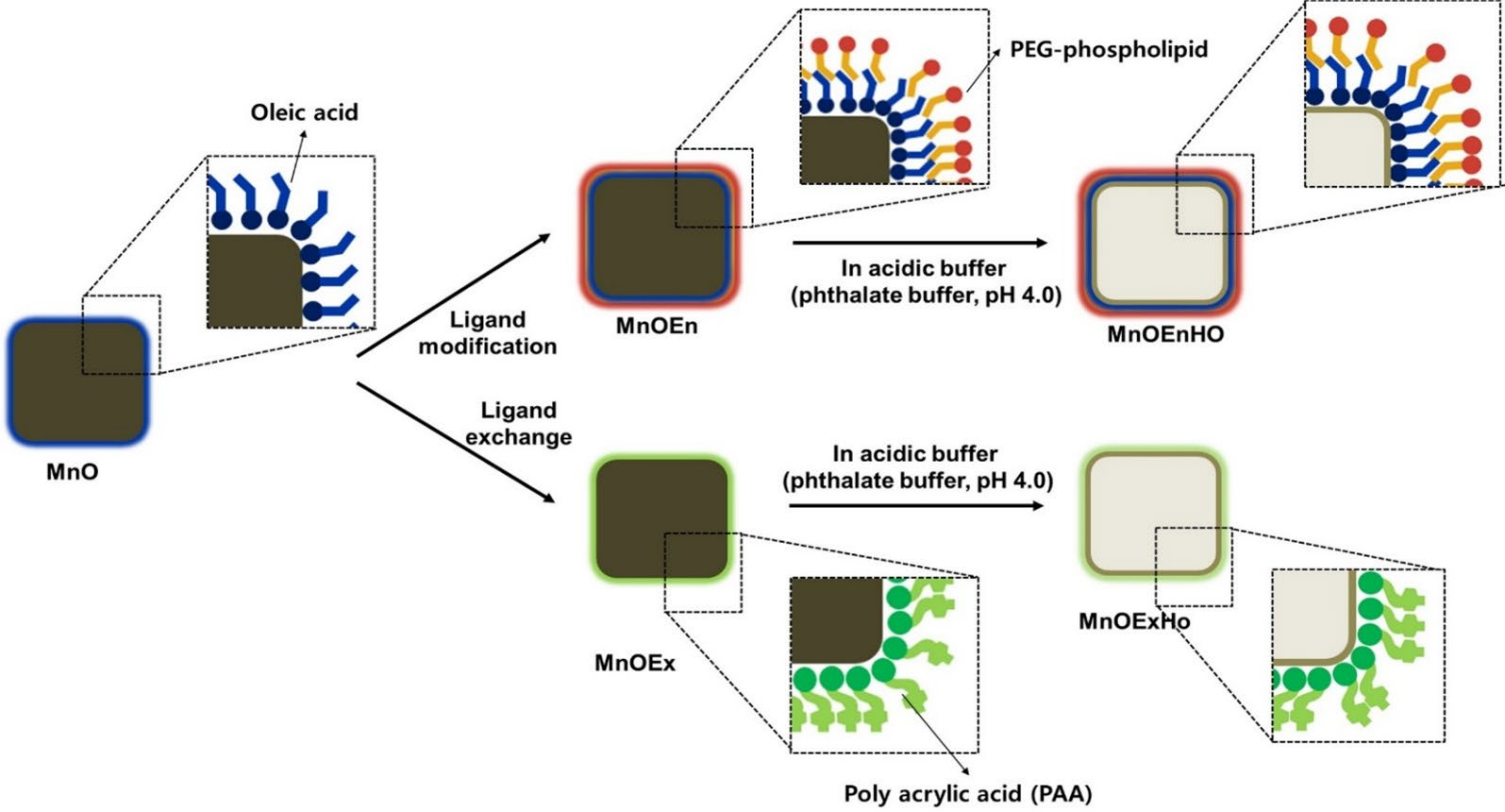
Schematic of the synthetic procedure of MnOEn, MnOEnHo, MnOEx and MnOExHo using MnO

Herein, we report on the development of four water-soluble MnO nanocubes, namely (1) MnOEn: poly(ethylene glycol) phospholipid-encapsulated MnO nanocubes (2) MnOEnHO: hollow poly (ethylene glycol) phospholipid-encapsulated MnO nanocubes (3) MnOEx: ligand (polyacrylic acid)-exchanged MnO nanocubes, and (4) MnOExHo: hollow ligand (polyacrylic acid)-exchanged MnO nanocubes. The ability to tailor MnO nanocubes for maximum contrast enhancement along with changes in their chemical and physical structure, MR properties, and biocompatibility has been demonstrated (Fig. 1).

## Results and discussion

### Synthesis and modification of MnO nanocubes

**Fig. 2:**
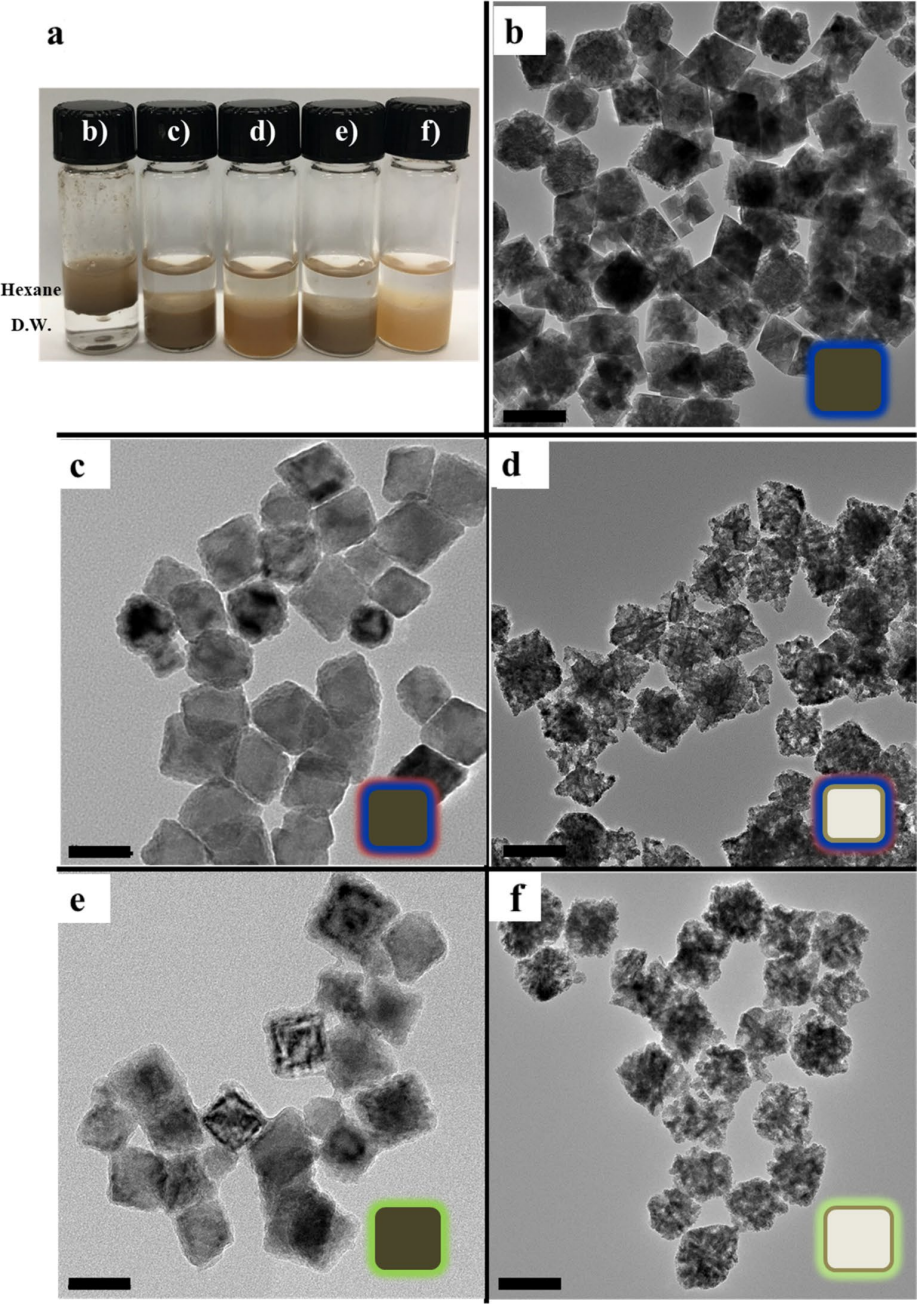
**a** Photos of the dispersibility of MnO in oil phase (hexane) and each MnO nanocube in aqueous phase (D.W.). TEM images of **b** MnO, **c** MnOEn, **d** MnOEnHo, **e** MnOEx, and **f** MnOExHo (scale bar: 50 nm)

Monodisperse MnO nanocubes were synthesized by the thermal decomposition of manganese oleate complexes at a high temperature in a nonpolar organic solvent. The TEM images of the as-prepared MnO nanocubes show that the nanoparticles exhibit uniform size of 70 nm (length, l) and 65 nm (width, w) with high monodispersity (Fig. 2b). Following the synthesis of the MnO nanocubes, surface modification was performed to transfer MnO into the aqueous phase (D.W.). The outer surface of the MnO nanocubes was modified either by surface encapsulation (MnOEn) using poly(ethylene glycol) (PEG)-phospholipid (Fig. 2c) or by ligand exchange (MnOEx) using polyacrylic acid (PAA) (Fig. 2e). In both cases, the nanoparticles were well-dispersed in the aqueous phase. Next, etching using an acidic solution (phthalate buffer, pH 4.0) was performed to form hollow MnO (MnOEnHo and MnOExHo) nanocubes (Fig. 2d, f). When the area around the MnO particles was acidic condition, the surface part was etched. In particular, it was known that in the phthalate buffer (pH = 4.0), the center of the particle was made hollow structure as the surroundings were etched [30, 31]. During etching, the particles formed a clear and light-brown solution, and the carved MnO core adhered itself to the magnetic bar. The entire process was simple and highly reproducible. The as-prepared MnO, MnOEn, MnOEnHo, MnOEx, and MnOExHo were characterized by HR-TEM (Fig. 2), which clearly shows that the synthesized nanoparticles retained the size and shape uniformity of the original MnO nanocubes. Furthermore, their successful phase transfer from an oil phase (hexane) to an aqueous solution (D.W.) was observed after surface ligand encapsulation or exchange from oleic acid to lipid or PAA, respectively (Fig. 2a).

### Characterization of MnO nanocubes

**Fig. 3:**
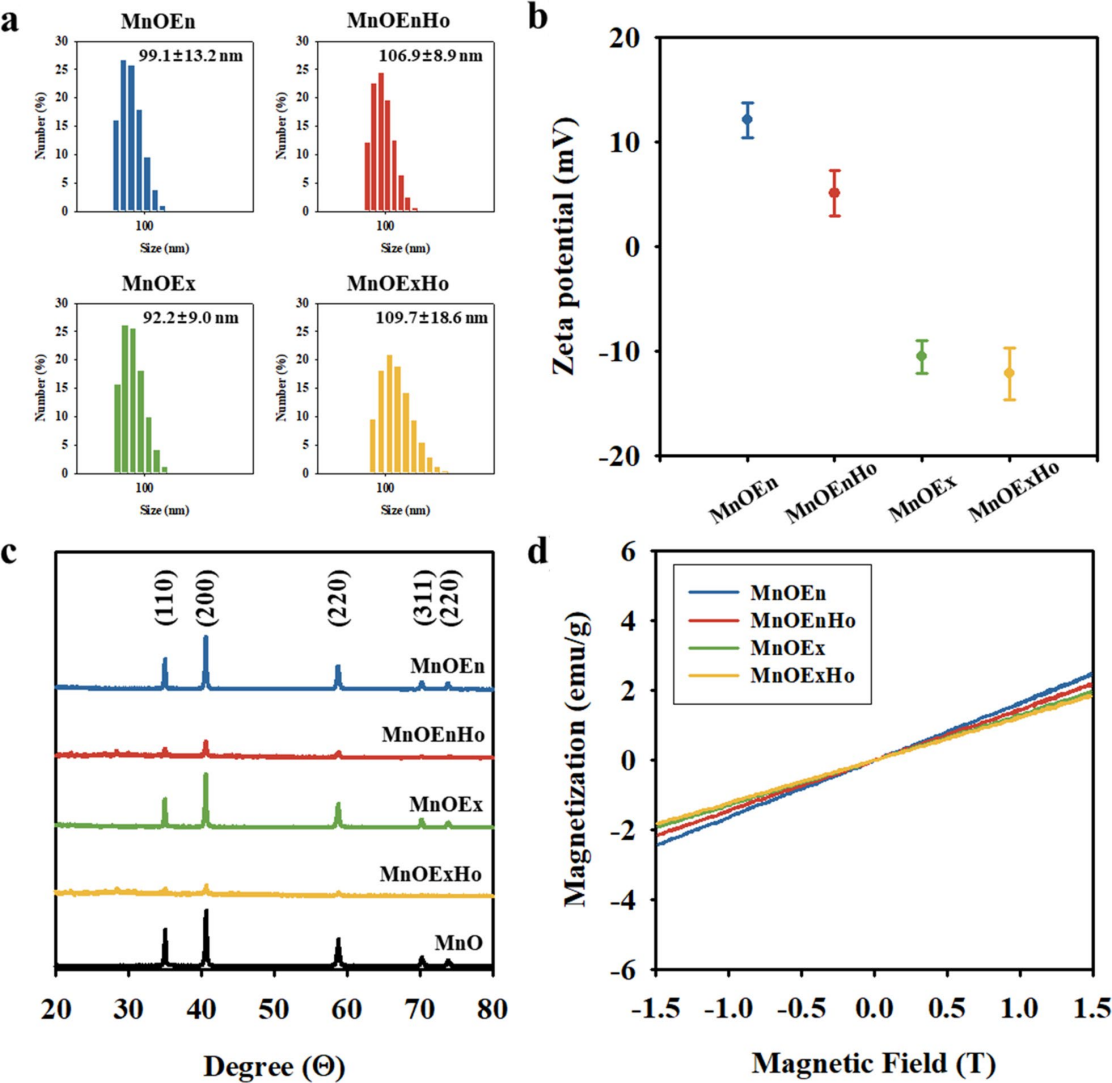
**a** Size distributions histogram using dynamic light scattering analysis, **b** Zeta potential values, **c** X-ray diffraction patterns, and **d** magnetic hysteresis loop analyzed using vibrating sample magnetometer [MnOEn (blue line), MnOEnHo (red line), MnOEx (green line), MnOExHo (yellow line), and MnO (black line)]

The size distribution of particles and their dispersion stability in the aqueous solution were measured using dynamic light scattering (DLS) analysis and zeta potential analyzer. DLS analysis defines the particles as spherical and measures the hydrodynamic diameter of the particles dispersed in the aqueous phase. Therefore, it is not accurate because it is in the form of a cube but its approximate hydrodynamic size and dispersibility in aqueous phase can be confirmed. There size were 99.1 ± 9.2 nm (MnOEn), 106.9 ± 8.9 nm (MnOEnHo), 92.2 ± 9.0 nm (MnOEx), and 109.7 ± 4.6 nm (MnOExHo), respectively (Fig. 3a). Especially, they exhibited low standard deviation, indicating that each particle were stably dispersed in the aqueous phase without aggregation. In addition, it was known that the surface charges were influenced by surface coating materials [40–44]. Therefore, the positive charges of MnOEn and MnOEnHo were attributed to the presence of amine groups in the lipid. Meanwhile, the negative charges of MnOEx and MnOExHo were attributed to the presence of carboxyl groups of PAA on their surfaces (Fig. 3b).

**Fig. 4:**
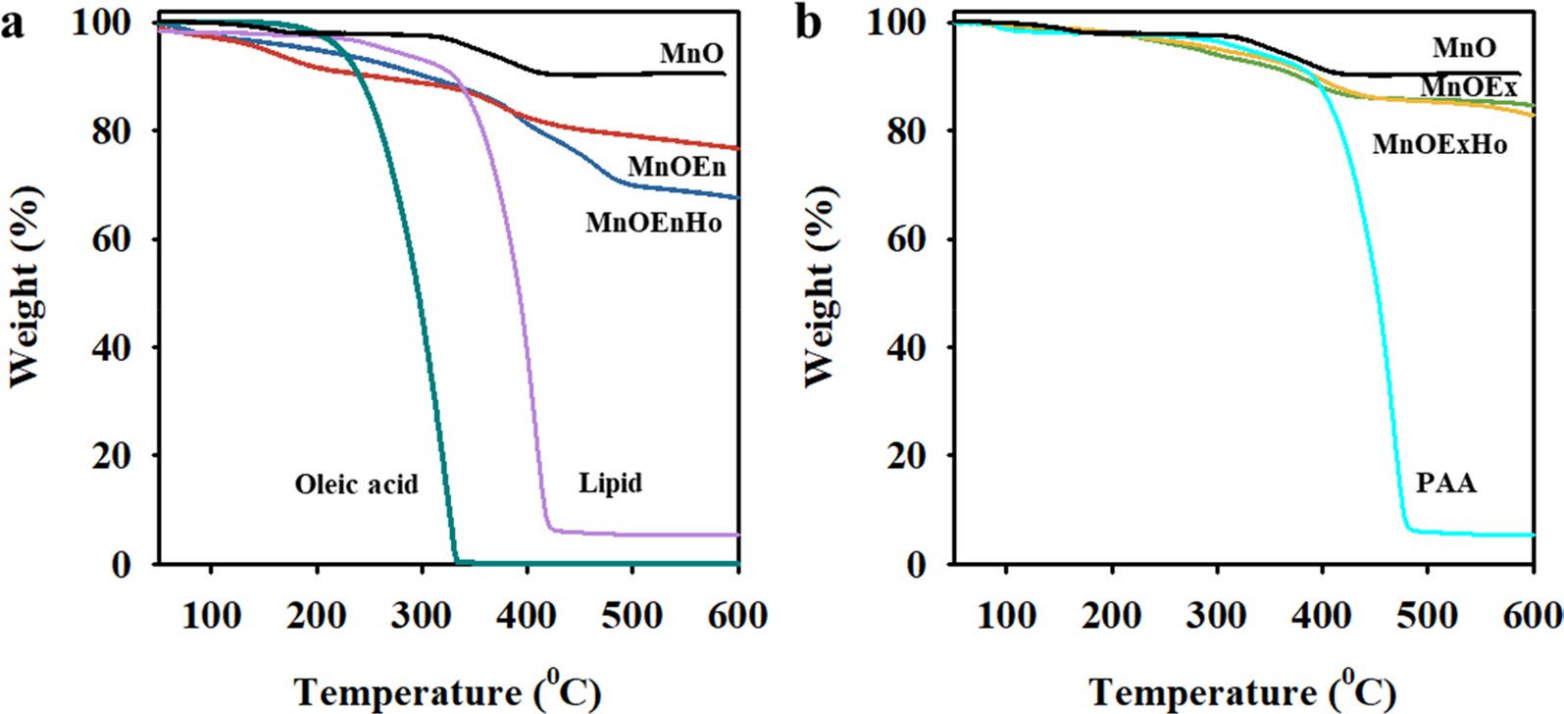
TGA analysis of **a** MnO (black line), MnOEn (blue line), MnOEnHo (red line), oleic acid (cyan line) and lipid (light violet line), **b** MnO (black line), MnOEx (green line), MnOExHo (yellow line) and polyacrylic acid (PAA) (light blue line)

The X-ray diffraction (XRD) results of MnO, MnOEn, MnOEnHo, MnOEx, and MnOExHo indicate that the crystalline peaks match the series of Bragg reflections corresponding to the standard, and the phase pure cubic rock salt structure of MnO (a = 4.442 Å) was maintained even after ligand exchange and surface modification (Fig. 3c). The field-dependent magnetization curves for MnOEn, MnOEnHo, MnOEx, and MnOExHo showed similar magnetization values (1.82–2.41 emu/mg) in an external magnetic field of 1.5 T with no remanence coercivity at zero field, indicating that these particles exhibited paramagnetic properties at room temperature (Fig. 3d). Thermogravimetric analysis (TGA) was used to confirm the ratio of surface modification on the surface of each MnO nanocube (Fig. 4). MnOEn contains 23.2 wt% of organic layer composed of oleic acid and PEG-phopholipid and 76.7 wt% of inorganic materials (MnO). After MnO etching, MnOEnHo was stably formed the hollow structure in MnO, so the ratio of organic layer in MnOEnHo increased to 32.4 wt% and the inorganic ratio decreased to 67.6 wt% (Fig. 4a). Likewise, MnOExHo had a less MnO ratio than MnOEx, indicating that the hollow structured nanocube was well formed (inorganic ratio in MnOEx: 84.8 wt% and inorganic ratio in MnOExHo: 83.1 wt%) (Fig. 4b).

**Fig. 5:**
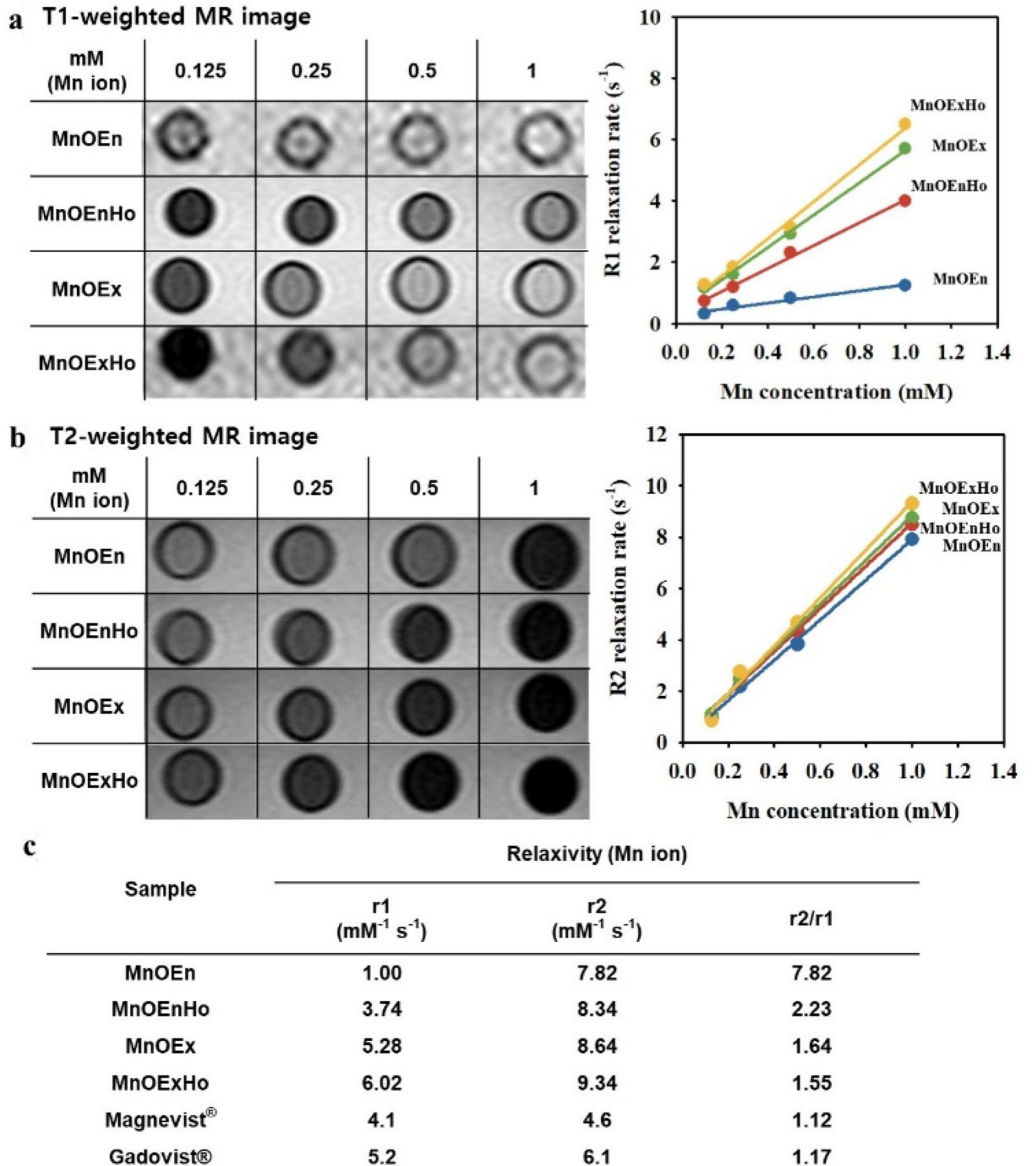
**a** T1-weighted MR images and **b** T2-weighted MR images depending on the concentration of each particle and plots of each relaxation (1/T1 and 1/T2) against Mn concentration. **c** Calculation of R1 relaxivity (r1), R2 relaxivity (r2) and r2/r1 [MnOEn (blue line), MnOEnHo (red line), MnOEx (green line), and MnOExHo (yellow line)]

In addition, we evaluated the feasibility of nanoparticles as T1 MRI agents and confirmed their MR contrast effects. The T1-weighted MR images acquired on a 3.0 T MRI scanner reveal the concentration-dependent signal enhancement, which validates the advantage of hollow MnO nanocubes over solid MnO nanocubes. As indicated in Fig. 5a, the MnOEn, MnOEnHo, MnOEx, and MnOExHo at the same concentration of 1 mM (based on the Mn concentration measured by inductively coupled plasma atomic emission spectroscopy) clearly showed bright signal enhancement. When we measured their MR signal intensities in an aqueous phase under various concentration at room temperature, the T-1 weighted MR images showed an increasingly stronger bright image as the concentration increased. Among them, MnOExHo showed the strong T1 MR signals, as the Mn concentration increased, and corresponding the longitudinal relaxivity (r1) was 6.02 mM^−1^ s^−1^. It was 6.02, 1.61, and 1.14 times those of MnOEn (1.00 mM^−1^ s^−1^), MnOEnHo (3.74 mM^−1^ s^−1^), and MnOEx (5.28 mM^−1^ s^−1^), respectively. As well, T1 relaxivity (r1) of MnOExHo was higher (approximately 1.16–1.47-fold) that that of commercial T1 MRI contrast media (Magnevist^®^: 4.1 mM^−1^ s^−1^ and Gadovist^®^: 5.2 mM^−1^ s^−1^). These enhancements in MnOExHo and MnOEnHo can be attributed to the increased volumetric capacity to access water molecules, which leads to an enhanced spin effect. The greater signal enhancement of MnOExHo, when compared to MnOEnHo, could be due to the bilayer effect of MnOEnHo, that is, the presence of a hydrophobic layer decreases the accessibility of water molecules to the metal ions. Furthermore, the T2-weighted MR images were obtained for each MnO nanocube concentration (Fig. 5b). The transversal relaxivity (r2) of MnOExHo, calculated on the basis of the Mn ion concentration, was 9.34 mM^−1^ s^−1^, which was 1.19, 1.12, and 1.08 times those of MnOEn (7.82 mM^−1^ s^−1^), MnOEnHo (8.34 mM^−1^ s^−1^), and MnOEx (8.64 mM^−1^ s^−1^), respectively (Fig. 5b). It has been reported that the ideal T1 MRI contrast agent should have an r2/r1 ratio close to 1 (one) [45, 46]. As shown in Fig. 5c, MnOExHo had high r1 value (6.02 mM^−1^ s^−1^) and r2/r1 value close to 1 (1.55), confirming the sufficient ability of MnOExHo as a T1 MRI contrast agent.

### Cell viability of MnO nanocubes

**Fig. 6:**
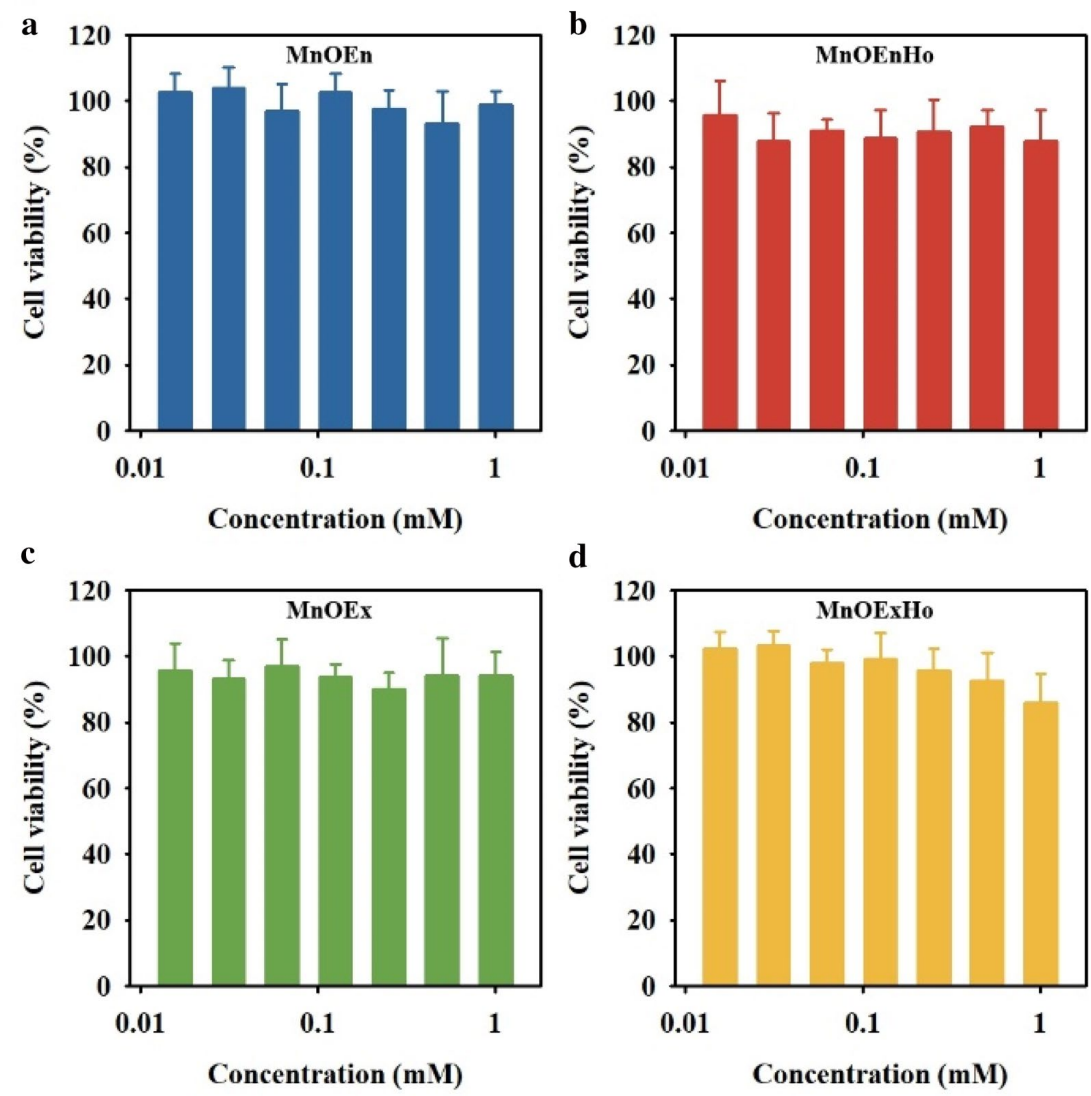
Cell viability of SNU-484 cells incubated with **a** MnOEn (blue), **b** MnOEnHo (red), **c** MnOEx (green) and **d** MnOExHo (yellow) at various concentrations for 24 h at 37 °C

The cellular cytotoxicity of the each MnO nanocubes was assessed in a gastric cancer cell line, SNU-484, using an MTT assay (3-(4,5-dimethylthiazol-2-y1)-2,5-diphenyltetrazolium bromide). As shown in Fig. 6, more than 80% of the cells survived at a high concentration of 1 mM all MnO nanocubes, indicating that these nanocubes showed biocompatibility even at high concentrations of MnO nanocubes.

### Cellular MR imaging using MnOExHo

**Fig. 7:**
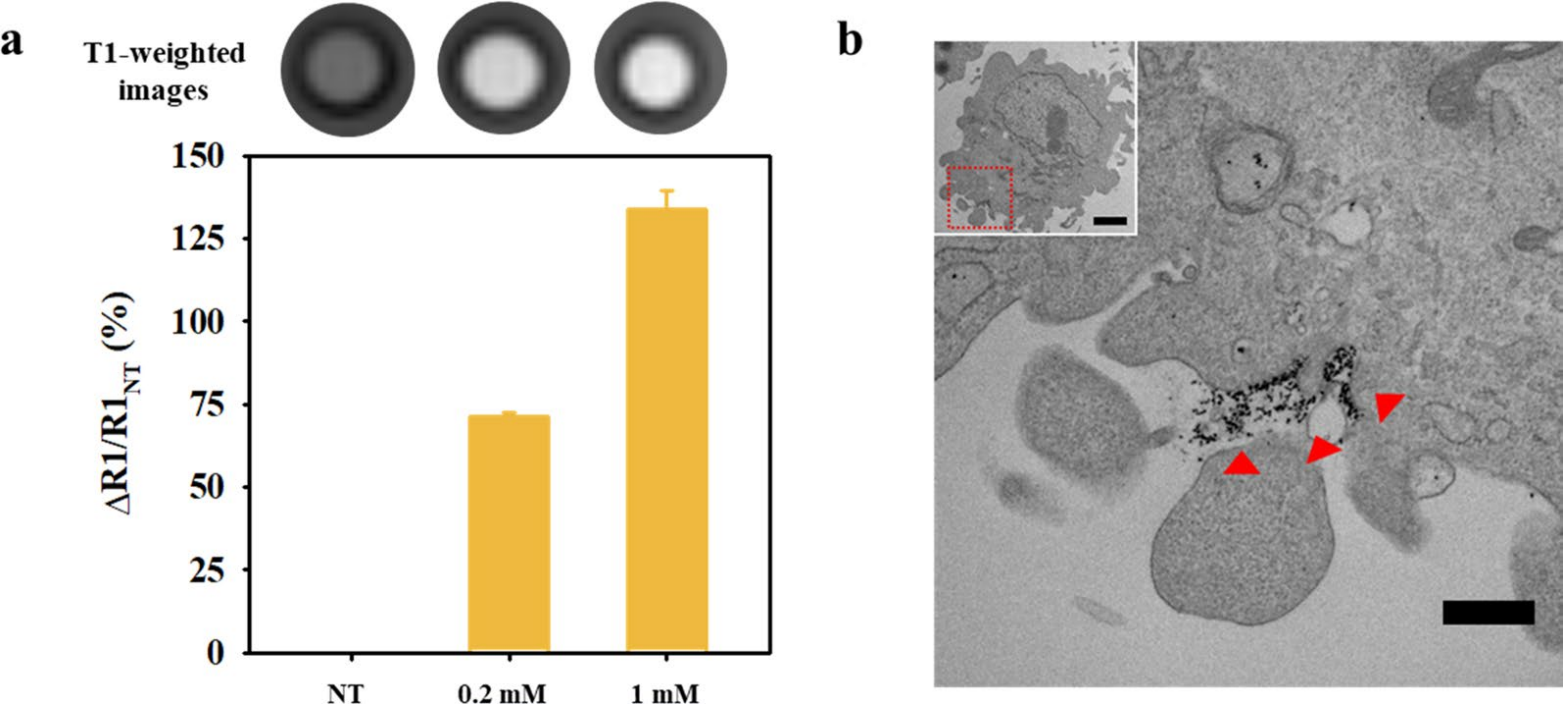
**a** T1-weighted MR images and relative signal intensity graphs of SNU-484 cells incubated with MnOExHo (ΔR1 = R1 − R1_NT_, NT: no treatment), **b** TEM images of SNU-484 cells treated with MnOExHo (scale bar: 500 nm, inset scale bar: 2 μm)

Additionally, MnOExHo having the highest T1 MRI sensitivity was treated with SNU-484 cells to evaluate its diagnostic ability as T1 MRI contrast agent. The SNU-484 cells treated with MnOExHo at concentrations of 0.2 and 1 mM showed significant MR contrast effects. MnOExHo-treated cells definitely showed bright images compared with the untreated cells. The T1-weighted MR images gradually changed from dark black to bright white as the concentration of treated MnOExHo in the cells increases (Fig. 7a). MR signal intensity (ΔR1/R1_NT_, ΔR1 = R1 − R1_NT_) was higher in treated cells than in non-treated cell (NT) (MnOExHo (0.2 mM)-treated cells: 71%, MnOExHo (1.0 mM)-treated cells: 134%) (Fig. 7a). In the TEM images of the MnOExHo-treated cells, furthermore, many black dots were observed, indicating the entry of the MnOExHo nanocubes into the SNU-484 cells (Fig. 7b).

### In vivo MR imaging of MnOExHo

**Fig. 8:**
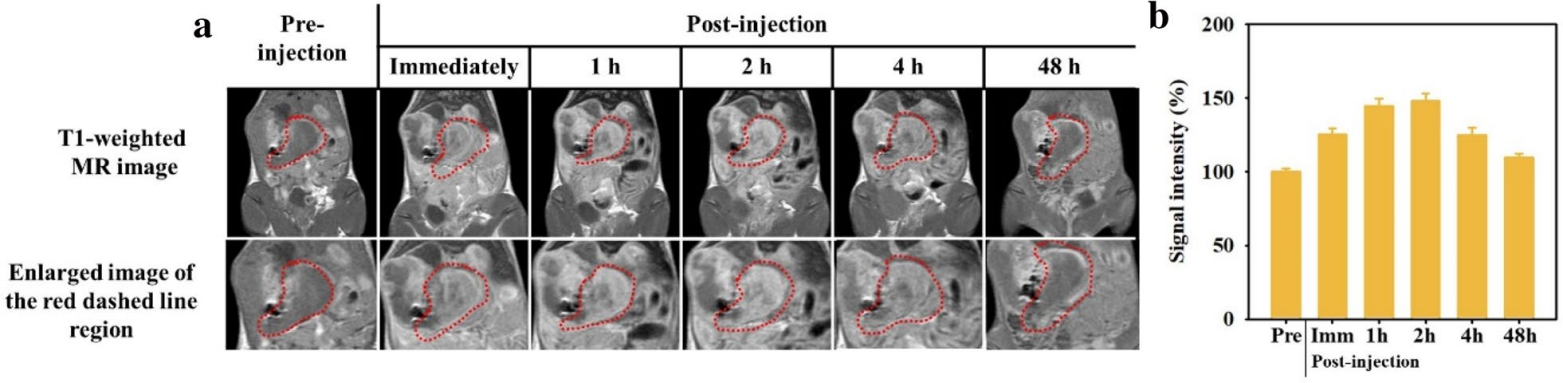
**a** In vivo T1-weighted MR images and **b** their relative T1 signal intensity (%) (red dashed line region) of orthotopic gastric cancer mice model after intravenous injection of MnOExHo (200 μg Mn per mouse) at different time points (pre-injection, immediately after, and 1, 2, 4, and 48 h post-injection)

We next performed in vivo MRI using MnOExHo to evaluate their diagnostic ability as a T1 MRI contrast agent in tumor-bearing mice. Tumor-bearing mice with gastric cancer were prepared, and MnOExHo (200 μgMn/μl) were injected into mouse tail vein (intravenous injection). We monitored MR imaging at different time intervals (pre-injection, immediately following injection, and 1, 2, 4, and 48 h post-injection) (Fig. 8), before and after MnOExHo injection. As soon as MnOExHo injection, tumor region [red dashed line region) appeared bright in the T1 MR images with high MR signal intensity, compared with before injection (Fig. 8a)]. To obtain quantitative and dependable results for signal intensity measurements, we analyzed the MR signal in the tumor by drawing ROIs of whole tumor volumes on both the T1-weighted images (Fig. 8b). The T1 signal intensity gradually increased after administration of MnOExHo, and showed the highest signal enhancement of 144.3% (1.44 times) 1 h after injection. Especially, MR images at 1–2 h after injection showed the brightest images at the tumor region, and MR signal intensities were also increased with a similar trend to MR images (Fig. 8). This is because MnOExHo circulated in the blood stream and gradually accumulated in tumor tissues by the enhanced permeability and retention (EPR) effect. After 48 h, no bright image of the tumor area was seen, and the increased MR signal was reduced as before particle injection (pre-injection). We judged that MnOExHo accumulated in tumor within 1–2 h and then excreted out of the tumor up to 48 h.

## Conclusions

In this study, we developed various types of MnO nanocubes for using effective T1-weighted MRI agents. The as-synthesized MnO nanocubes were rendered water-soluble by ligand exchange and ligand encapsulation. Moreover, treatment with the acidic solution (phthalate buffer) was used to form hollow MnO nanocubes. Furthermore, it was confirmed that the hollow structure and the surface ligand exchange of the MnO nanocubes allow greater access of Mn ions to the water molecules, thus exhibiting a maximum enhancement of longitudinal relaxation time and inducing strong T1 MR signal. Therefore, this study suggest that MnOExHo could be used as effective T1 contrast agents for biomedical research.

## Experimental methods

### Materials

Diethylene glycol (DEG), manganese (II) formate [Mn(COOH)_2_], tri-n-octylamine (TOA), oleic acid (OA), and polyacrylic acid (PAA) were purchased from Sigma-Aldrich. Ethanol, chloroform and a phthalate buffer (pH 4.0) were procured from Samchun Chemical. 1,2-distearoyl-sn-glycero-3-phosphoethanolamine-*N*-[methoxy(aminoethylene glycol)-2000] (PEG-Phospholipid) (mPEG-2000 PE, Avanti Polar Lipids, Inc.) was used as-purchased without any purification. All other reagents purchased from commercial sources were used as-obtained without further purification. Furthermore, ultrapure deionized water was used for all the synthesis processes.

### Synthesis of MnO nanocubes

Mn(COOH)_2_ (1 mmol) was added to a mixture containing 3 mmol of TOA and 3 mmol of OA. The resulting mixture was heated to 130 °C under vacuum until all the moisture was removed. First, the solution was heated to 280 °C for 1 h and then refluxed at 330 °C, where its color changed from brownish red to green. Then, it was left for 1 h under N_2_ flow to yield uniform MnO nanocubes. Finally, it was allowed to cool at room temperature (25 °C). The resulting particles were precipitated by adding ethanol and centrifuged at 6000 rpm for 10 min; they were then washed with chloroform and ethanol twice and finally, dispersed in chloroform.

### Fabrication of MnOEn and MnOEx

A total of 10 mg of organic dispersible MnO nanocubes was added to 10 mL of chloroform solution containing 50 mg of PEG-phospholipid in a ratio of 5:1. After evaporating the solvent, the solution was incubated at 80 °C in vacuum for 1 h. The addition of 20 mL of water resulted in a clear, dark-brown suspension. Next, centrifugation was performed at 21,000 rpm for 30 min, and the particles were ultimately dissolved in the aqueous phase. Ligand exchange reactions were performed to modify the surface of the hydrophobic oleate-capped MnO nanocubes. A solution containing 20 mL of DEG and 200 mg of PAA was heated to 200 °C under vacuum for 1 h with magnetic stirring, followed by refluxing under N_2_ for 2 h at the same temperature. Then, a solution of 40 mg MnO in 4 mL of hexane was immediately added to the flask, and the resulting mixture was maintained at 200 °C for another 6 h. After cooling the solution to room temperature, the resulting nanocubes were precipitated by adding ethanol and collected by centrifugation. The particles were ultimately dissolved in the aqueous phase.

### Preparation of hollow-typed MnO nanocubes (MnOEnHo and MnOExHo)

A total of 10 mg of the synthesized MnOEn and MnOEx nanocubes was dispersed into 20 mL of phthalate buffer at pH 4.0 and stirred for 12 h to carve away the MnO core. The resulting particles (MnOEnHo and MnOExHo) were obtained by centrifugation and resuspension in distilled water.

### Cell cytotoxicity assay

SNU-484 cells from a gastric cancer cell line were obtained from the Korean Cell Line Bank (Seoul, Korea). The cells were maintained in RPMI medium supplemented with 10% FBS and 1% antibiotic–antimitotic at 37 °C with 5% CO_2_. The in vitro cytotoxicity of each MnO nanocube was assessed by the standard MTT assay. The SNU-484 cells were seeded in a 96-well plate at a density of 10^4^ cells per well, and cultured in 5% CO_2_ at 37 °C for 24 h. Then, various concentrations of each MnO nanocube were added to the medium and the cells were further incubated for 24 h. Afterward, the media was removed and MTT1 solution was added; the cells were incubated for another 4 h. The medium was then replaced with MTT2 solution, and, after 24 h, measurements were recorded at an absorbance–wavelength of 575 nm and a reference wavelength of 650 nm, using a multimode microplate reader. The cell viabilities were determined by calculating the ratio of the intensity of purple formazan formed in the viable cells treated with the nanocubes to the intensity of that in the untreated control cells.

### Cellular internalization of MnOExHo nanocubes

First, 2 × 10^7^ SNU-484 cells suspended in PBS (1 mL) was incubated with MnOEnHo (14 μg and 70 μg, respectively) and additionally incubated for 4 h at 37 °C. MnOEnHo incubated SNU-484 cells were e-suspended in 200 μL of 4% paraformaldehyde for MR imaging analysis. Their relaxivity coefficient (mM^−1^ s^−1^) was equal to the ratio of R1 (1/T1, s^−1^) and the concentration of Mn. As well, the cellular internalization of the MnOExHo nanocubes was confirmed by TEM (JEOL-1011). SNU-484 cells (10^6^ cells/well) were seeded onto six-well plates overnight and then incubated with the MnOExHo nanocubes (1 mM in medium) in a 5% CO_2_ atmosphere at 37 °C. After incubation for 8 h, the SNU-484 cells treated with the MnOExHo nanocubes were washed thrice with a phosphate buffered solution (pH 7.4, 10 mM), trypsinized (0.5 mL), and subsequently, harvested. Then, following collection, the cells were fixed using the standard fixation and embedding protocol for resin-section transmission electron microscopy (TEM) and sectioned using a LEICA Ultracut UCT ultra-microtome (Leica Microsystems, Austria).

### In vivo model procedure

All animal experiments were conducted with the approval of the Association for Assessment and Accreditation of Laboratory Animal Care International. To establish an orthotopic mouse model of gastric cancer, SNU-484 cells (1.0 × 10^7^ cells) were implanted into the fundic glands of the stomachs of male mice (6-week-old balb/c-nude mice). Then, MRI was performed on five mice, 4 weeks after the tumor cell transplantation. When the tumor size reached approximately 500 mm^3^, water-soluble MnOExHo (200 μg) was injected intravenously into the tail vein.

### MR imaging procedure

We performed MR imaging experiment of MnO nanocubes solution with a 3.0 T clinical MRI scanner with a micro-47 surface coil (Intera; Philips Medical Systems, Best, the Netherlands). The R1 relaxivity of various concentrations of MnO nanocubes was measured by the Carr–Purcell–Meiboom–Gill (CPMG) sequence at room temperature with the following parameters: echo time (TE) = 60 ms, repetition time (TR) = 4000 ms, slice thickness = 2.0 mm, number of acquisitions = 1, and point resolution = 234 × 234 μm^2^. The relaxivity values of R1 were calculated by a series of T1 values, when plotted as 1/T1 versus [Mn]. The relaxivity coefficient (mM^−1^ s^−1^) was equal to the ratio of R1 (1/T1, s^−1^) and the nanoparticle concentration. In vitro/in vivo MRI experiments were performed using a 3.0 T clinical MRI instrument equipped with a micro-47 surface coil (Intera; Philips Medical Systems, Best, Netherlands). To acquire cellular T1-weighted MR images, following parameters were adopted: resolution = 234 × 234 mm, section thickness = 3.0 mm, TE = 18 ms, TR = 625 ms, and number of acquisitions = 2. For T1-weighted MR images of the nude mice, the following parameters were adopted: resolution = 234 × 234 mm, section thickness = 2.0 mm, TE = 60 ms, TR = 4000 ms, and number of acquisitions = 1.

## Data Availability

All data generated or analyzed during this study are included in this published article and its additional file.

## Abbreviations

MnO: Manganese oxide
En: Encapsulation
Ex: Exchange
Ho: Hollow
MRI: Magnetic resonance imaging
DEG: Diethylene glycol
TOA: Trioctylamine
OA: Oleic acid
PAA: Polyacrylic acid
